# Comparative Proteomics Analysis of *Anisakis simplex* s.s.—Evaluation of the Response of Invasive Larvae to Ivermectin

**DOI:** 10.3390/genes11060710

**Published:** 2020-06-26

**Authors:** Iwona Polak, Elżbieta Łopieńska-Biernat, Robert Stryiński, Jesús Mateos, Mónica Carrera

**Affiliations:** 1 Department of Biochemistry, Faculty of Biology and Biotechnology, University of Warmia and Mazury in Olsztyn, 10-719 Olsztyn, Poland; iwona.polak@uwm.edu.pl (I.P.); robert.stryinski@uwm.edu.pl (R.S.); 2 Department of Food Technology, Marine Research Institute (IIM), Spanish National Research Council (CSIC), 36-208 Vigo, Spain; jmateos@iim.csic.es

**Keywords:** parasitic nematode, anisakiasis, *Anisakis simplex*, proteome, mass spectrometry, real-time PCR, antiparasitic drugs

## Abstract

Ivermectin (IVM), an antiparasitic drug, has a positive effect against *Anisakis simplex* s.s. infection and has been used for the treatment and prevention of anisakiasis in humans. However, the molecular mechanism of action of IVM on *A. simplex* s.s. remains unknown. Herein, tandem mass tag (TMT) labeling and extensive liquid chromatography coupled with tandem mass spectrometry (LC-MS/MS) analysis were used to identify the effect of IVM on the proteome of *A. simplex* s.s. *in vitro*. During the study, 3433 proteins, of which 1247 had at least two protein unique peptides, were identified. Comparative proteomics analysis revealed that 59 proteins were differentially regulated (DRPs) in IVM-treated larvae, of which 14 proteins were upregulated and 38 were downregulated after 12 h of culture, but after 24 h, 12 proteins were upregulated and 22 were downregulated. The transcription level of five randomly selected DRPs was determined by real-time PCR as a supplement to the proteomic data. The functional enrichment analysis showed that most of the DRPs were involved in oxidoreductase activity, immunogenicity, protein degradation, and other biological processes. This study has, for the first time, provided comprehensive proteomics data on *A. simplex* s.s. response to IVM and might deliver new insight into the molecular mechanism by which IVM acts on invasive larvae of *A. simplex* s.s.

## 1. Introduction

Anisakiasis is a zoonotic disease triggered by the third-stage larvae of nematodes from the genus *Anisakis*. This parasite habitually parasitizes adult marine mammals. Intermediate and/or paratenic hosts of the larvae are crustaceans, cephalopods, and fish [1]. Humans are an accidental host of this parasitic nematode. Infection occurs upon consumption of raw or undercooked marine fish and crustaceans contaminated with the third-stage larvae of *A. simplex* [2]. Infected dishes of raw fish, such as sushi and sashimi, as well as the consumption of marinated or raw fish in European countries such as Italy and Spain, are significant sources of the infection [3]. Greater awareness of *Anisakis* infection has resulted in an increase in the frequency of reported anisakiasis cases in many more countries, besides the expected ones (Italy, Spain, Japan), e.g., in Poland the first case of this disease was described in 2020 [4,5,6,7,8].

Two main symptoms of anisakiasis are described: inflammatory reactions and direct tissue damage as a result of the penetration of the invasive larvae into the target organ site [9,10,11]. The survival time of *Anisakis* in humans is very short; usually, larvae become expelled or destroyed by the host immune system in a few days, up to two weeks [12]. Invasion of the intestinal wall by the parasite sometimes results in the development of eosinophilic granuloma or perforation, which causes direct tissue damage [10].

However, there are no adequate methods of diagnosis or estimating the occurrence of this disease. In addition, the mechanism of action of anthelmintics on this parasitic nematode is not known. This indicates a serious problem, and clarifying the activity of parasite-specific proteins on anthelmintics may contribute to a reduction in the need for and/or more effective administration of drugs. Routinely used anthelmintics have a broad spectrum of action, and finding target proteins involved in the interaction with, e.g., ivermectin (IVM) might be a necessary preliminary step to identifying the specific mechanism of drug metabolism.

IVM is a member of the avermectin family of compounds, a class of macrocyclic lactones with insecticidal properties, and is one of the most extensively used antiparasitic agents worldwide [13]. IVM is the most studied member of this family and has been considered one of the most successful discoveries in the fight against infections caused by parasitic roundworms. IVM is highly hydrophobic and deeply inserts itself into the subunit interfaces of transmembrane domains of Cys-loop receptor family members [14]. Ivermectin’s primary targets are glutamate-gated chloride channels, although it is also active against other invertebrate neurotransmitter receptors, including GABA, histamine-gated chloride channels, pH-gated chloride channels [15,16,17], glycine receptors, and α7-nicotinic acetylcholine receptors (nAChR) [18,19]. A deciding factor in the combined lethality in nematodes and safety in mammalian hosts is the differential potency with which ivermectin activates these receptors. For example, a glutamate-gated chloride channel receptor is typically activated by low nanomolar (<20 nM) concentrations [20,21], whereas GABA_A_R and the glycine receptor chloride channel require concentrations in the low micromolar (1–10 μM) range [18]. At several other receptors, ivermectin activates no response but modulates the responses to the neurotransmitter agonist. These include some invertebrate GABA_A_R and the human α-7 nAChR [14]. 

Ivermectin affects the feeding, motility, and reproduction of nematodes [22]. It was increasingly noted that the rapid microfilarial clearance following ivermectin dosing results not from the direct impact of the drug but via suppression of the parasite’s ability to evade the host’s natural immune defense mechanisms [23,24]. Immunomodulatory agents often display fewer side effects than drugs, as well as producing less opportunity for the creation of resistance in target microorganisms, which helps explain the absence of drug resistance in humans [25]. Ivermectin, as a drug with many functions such as anticancer [26] and antiviral, including against SARS-CoV-2 [27], is becoming more and more puzzling, and discovering its many as yet unknown functions can be a source of inspiration in scientific research.

One approach that is being used to characterize the proteins expressed by the parasites (proteome) is quantitative proteomics. Significant progress has been made in overcoming the technical hurdles faced when characterizing the parasite proteins. The challenges are now to use this information to investigate the parasite attributes such as virulence, antigenicity, and drug resistance and to translate the findings from these studies to produce human and animal health benefits. Proteomic studies not only give insight into the description of particular pathology but have the potential to precisely identify drug targets, which is necessary because strains resistant to anthelmintic drugs have already been described [28,29,30]. As already mentioned, the main control strategy for both human and animal parasitic nematodes is antiparasitic drugs [31]. Nonetheless, reliance on a limited number of anthelmintic drugs to control humans’ helminthiases is not ideal, and additional complementary drugs and control strategies are required. In addition, there is a need for a detailed understanding of the mode of action of the drugs currently available in order to maximize their effective lifetime. Proteomic studies have begun to describe the action of these drugs as well as to identify potential new drug targets [32]. The availability of multiple parasite genomes for comparative analysis and the application of high-throughput sequencing technologies to classical genetic and proteomic approaches may provide answers to these questions soon. Comparative proteomics studies have increased our understanding of the action of proteins taking part in drug metabolism. Proteome studies of *A. simplex* s.s. L3 and L4 larvae carried out previously showed the possibility of using high-throughput techniques for targeted analyses [33].

In the present study, we applied TMT-based quantitative proteomics to *A. simplex* s.s. cultured in vitro with and without IVM to indicate proteins with a potential role in parasite response to the drug. In addition, differentially regulated proteins (DRPs) were identified not only between the control and IVM-treated samples, but also between different culture times, which were applied to complement the results and to pinpoint important differences according to the period of IVM action. Our study, besides describing proteins modulated by IVM in the *A. simplex* s.s., demonstrates that the TMT-based proteomic approach in parasitological studies offers an effective way of determining which proteins or pathways should be targeted for therapeutic purposes.

## 2. Materials and Methods

### 2.1. Anisakis Simplex

The experiment was performed on live *A. simplex* s.s. L3 larvae, obtained from herring *Clupea harengus membras* caught in the coastal waters of the southern Baltic Sea.

Ten of the larvae used in the experiment were subjected to taxonomic identification based on the amplification of the ITS1/ITS2 region of genomic DNA, isolated via the use of Xpure™ Cell & Tissue micro (A & A Biotechnology, Gdynia, Poland) according to the manufacturer’s instructions. The identification was done with the use of an Anis Sensitive Sniper Real-Time PCR kit according to the manufacturer’s protocol (A & A Biotechnology, Gdynia, Poland). The kit was used to identify representatives of nematodes from the Anisakidae family found in the Baltic and North Atlantic (*Anisakis simplex* s.s., *A. pegreffii*, *Pseudoterranova decipiens*, *P. krabbei*, *Contracaecum osculatum*, and *Hysterothylacium aduncum* (a species of the Raphidascarididae family)).

### 2.2. In Vitro Culture with Ivermectin

Harvested parasites were cleaned of all impurities and washed several times in sterile 0.9% NaCl. In vitro culturing of *A. simplex* L3 larvae was carried out according to Iglesias et al. [34] in RPMI-1640 medium (R8758, Sigma Aldrich, Poznan, Poland) enriched with fetal bovine serum (Sigma Aldrich, F7524) and pepsin (Sigma Aldrich, P7125, Poznan, Poland). The medium was acidified to pH = 4 with 1M HCl. A total of 3 mL of medium was pipetted into each well of the six-well culture plate (from BD Biosciences, Warsaw, Poland). The experiment was conducted in the incubator under anaerobic conditions, at 37 °C and 5% CO_2_, with the addition of ivermectin (IVM) (Sigma Aldrich, I8898, Poznan, Poland) in 0.1% DMSO solution (Sigma Aldrich, D8418, Poznan, Poland ) at a concentration of 12.5 µg/mL of culture medium according to Hu et al. [35]. Five parasites were placed in each well (45 in total). The *A. simplex* L3 larvae with no drug were cultured as a control (three samples; five larvae in each sample). Larvae cultured with IVM (all were alive after the treatment) were taken from the cultures after 12 h (three samples; five larvae in each sample) or 24 h (three samples; five larvae in each sample) and were preserved at −80 °C until the next step of the analysis.

### 2.3. Samples Preparation

Protein extracts were obtained from L3 *A. simplex* larvae. For that, parasites were crushed manually with a sterile plastic pestle in 2 mL centrifuge tubes. Then, protein extraction was performed in 1.5 mL of lysis buffer (60 mM Tris-HCl pH 7.5, 1% lauryl-maltoside, 5 mM PMFS, and 1% DTT), as described by Stryiński et al. [33]. The protein concentration was quantified using the bicinchoninic acid method (Pierce BCA Protein Assay Kit, Thermo Fisher Scientific, Waltham, MA, USA).

Then, the SDS-PAGE electrophoresis was performed as a control step to determine whether the protein extraction was done correctly. Protein extracts were separated and evaluated on 12% polyacrylamide gel (acrylamide/bis-acrylamide, 20:1) with a stacking gel of 4% polyacrylamide, as described by Stryiński et al. [33]. Running conditions were 80 V for the first 20 min and then 120 V until the end of the electrophoresis. Gels were silver stained using the Pierce Silver Stain for Mass Spectrometry kit (Thermo Fisher Scientific, Waltham, MA, USA) (Figure S1).

### 2.4. Protein Precipitation and Trypsin Digestion

A total of 100 μg of the protein from each sample was transferred into a new tube and methanol/chloroform precipitation was performed as described by Carrera et al. [36]. Then, ultrafast tryptic digestion with the simultaneous application of high-intensity ultrasound (HIFU) was carried out, as described previously by Stryiński et al. [33].

### 2.5. TMT Labeling and Reversed-Phase Fractionation

TMT 10-plex isobaric label reagents (0.8 mg, Thermo Fisher Scientific, Waltham, MA, USA) were resuspended in 41 μL of anhydrous acetonitrile and added to 100 μg of protein digest, as described by Stryiński et al. [33]. Within the experiment, samples were labeled with TMT10-plex in triplicate (L3 larvae treated with IVM for 12 h: 126, 127N, 127C; L3 larvae treated with IVM for 24 h: 128N, 128C, 129N; L3 larvae with no drug as a control: 129C, 130N, 130C). Samples were combined in a new tube at equal amounts according to the manufacturer’s instructions. The reaction was carried out for 1 h at room temperature, as described by Stryiński et al. [33]. The TMT-labeled peptide concentration was measured using a Pierce Quantitative Colorimetric Peptide Assay (Thermo Fisher Scientific, Waltham, MA, USA). To increase the number of peptide identifications, labeled samples were fractionated using a Pierce High-pH Reversed-Phase Peptide Fractionation Kit (Thermo Fisher Scientific, Waltham, MA, USA) following the manufacturer’s instructions. A total of 100 μg of the labeled sample was dissolved in 300 μL of 0.1% TFA solution. Eight different sample fractions were obtained stepwise, using the appropriate elution solutions according to the kit manufacturer’s instructions. The peptide content of each fraction was determined by colorimetric analysis using the Quantitative Colorimetric Peptide Assay (Thermo Fisher Scientific, Waltham, MA, USA) and evaporated to dryness using vacuum centrifugation (SpeedVac concentrator, Thermo Fisher Scientific, Waltham, MA, USA). The samples (eight fractions plus the wash and flow throughput) were stored at −80 °C until further analysis.

### 2.6. LC-MS/MS Analysis

Peptide samples were acidified with 0.1% formic acid, cleaned on a C_18_ MicroSpin™ column (The Nest Group, Southborough, MA, USA), and analyzed by LC-MS/MS using a Proxeon EASY-nLC II liquid chromatography system (Thermo Fisher Scientific, Waltham, MA, USA) coupled to a LTQ-Orbitrap Elite mass spectrometer (Thermo Fisher Scientific, Waltham, MA, USA). Peptide separation (1 μg) was done as described by Stryiński et al. [33]. In brief, a spray voltage of 1.95 kV and a capillary temperature of 275 °C were used for ionization. The peptides were analyzed in a positive mode from 400–1600 amu (1 μscan), followed by 10 data-dependent HCD MS/MS scans (1 μscans), using a normalized collision energy of 38% and an isolation width of 1.5 amu. Dynamic exclusion for 30 s after the second fragmentation event was applied, and unassigned charged ions were excluded from the analysis.

### 2.7. Processing of the MS/MS Data

All acquired MS/MS spectra were analyzed using SEQUEST-HT (Proteome Discoverer 2.4 package, Thermo Fisher Scientific, Waltham, MA, USA) against a custom-made database containing protein entries for *A. simplex* plus “Nematoda”, available in the UniProt/TrEMBL database. The following restrictions were used: full tryptic cleavage with up to two missed cleavage sites and tolerances of 7 ppm Da for parent ions and 0.6 Da for MS/MS fragment ions. TMT-labeling (lysine and peptide n-terminus) and carbamidomethylation of cysteine were set as fixed modifications. The permissible variable modifications were set as described by Stryiński et al. [33].

### 2.8. Statistical Analysis

The results were subjected to statistical analysis to determine the peptide false discovery rate (FDR) using a decoy database and the Target Decoy PSM Validator algorithm [37]. The FDR was kept below 1%, and for further analysis, only proteins quantified with at least two unique peptides were submitted. Relative quantification was performed using the Quantification Mode and normalization against total peptide amount (Proteome Discoverer 2.4 package, Thermo Fisher Scientific, Waltham, MA, USA). After relative quantification, several filters were applied to the results to obtain the final list of differentially expressed proteins: (a) at least a 1.5-fold change in control/12 h, control/24 h, and 24 h/12 h normalized ratios; (b) Kruskal‒Wallis one-way analysis of variance with *p*-value ≤ 0.05.

### 2.9. Functional Analysis

To perform functional enrichment analysis, the final list of nonredundant protein IDs obtained after relative quantification (1247) was classified into three different categories of Gene Ontology (GO): biological processes, cell components, and molecular functions. GO analysis was performed using g:GOSt, the core of the g:Profiler (ELIXIR, Hinxton, Cambridgeshire, UK) that performs statistical enrichment analysis [38] (https://biit.cs.ut.ee/gprofiler/gost). The g:GOSt web-based tool applied an overrepresentation test controlled with the g:SCS algorithm, the default method for computing multiple testing correction for *p*-values gained from GO enrichment analysis. It corresponds to an experiment-wide threshold of a = 0.05, i.e., at least 95% of matches above the threshold are statistically significant.

The significantly enriched functional GO categories were reported by comparing the input data with the background of GO annotations for parasite-specific data from WormBase ParaSite (*Anisakis simplex* PRJEB496).

### 2.10. Network Analysis

Network analysis was performed by submitting the DRPs dataset to STRING (v. 10.5; ELIXIR, Hinxton, Cambridgeshire, UK) (Search Tool for the Retrieval of Interacting Genes) (https://string-db.org/). Interactions have been identified through comparing the input data with the background of the *Caenorhabditis elegans*, the phylogenetically closest nematode, available in the STRING database. Network clustering was applied using the inflation parameter MCL = 3. 

### 2.11. Transcription Level Determination by Real-Time PCR

The transcription level (mRNA) of five DRPs was determined by real-time PCR. The primers for the chosen genes were designed using the Primer 5.0 software and listed in Table S1. The elongation factor 1-alpha (*ef1-α*) gene was used as the endogenous reference gene [39]. The total RNA of larvae was isolated using a Total RNA Mini Kit (A & A Biotechnology, Gdynia, Poland). The total cDNA was produced using 1 μg of total RNA, oligo(dT)18 primer, and the MMLV-RT reverse transcriptase (20 U/µL) (TranScriba Kit; A & A Biotechnology, Gdynia, Poland) according to the manufacturer’s protocols. The real-time PCR reaction mixture contained 1 μL of cDNA, 5 μL of 2 × SYBR RT PCR MIX SYBR B (A & A Biotechnology, Gdynia, Poland), 0.25 μL of each primer, 0.25 μL of Rox Reference Dye II (A & A Biotechnology, Gdynia, Poland), and 3.25 μL of nuclease-free water to a final volume of 10 μL, according to the manufacturer’s protocols. The real-time PCR reactions were performed on a 7500 Fast Real-Time PCR thermocycler (Applied Biosystems, Foster City, CA, USA). The data, presented as the fold change in gene expression normalized to an endogenous reference gene *ef1-α* and relative to the untreated control (relative quantification RQ = 1), were calculated using the comparative Pfaffl method [40]. Every gene expression was determined six times.

Data were expressed as means ± standard deviations using one-way ANOVA in Prism 7 software (GraphPad Software Inc., San Diego, CA, USA). Differences between means were assessed by Dunnett’s multiple comparison test. *p*-values below 0.1234 (nonsignificant) were considered statistically significant (0.0332 (*), 0.0021 (**), 0.0002 (***), and <0.0001 (****)).

## 3. Results and Discussion

The main control strategy for human parasites is through anthelmintic drugs. The metabolic processes activated in parasites during the host drug treatment are still not fully understood. In addition, there is a need for a detailed understanding of the mode of action of the drugs currently available in order to maximize their effective lifetime [32]. Proteomics might usefully contribute to an understanding of drug metabolism mechanisms and resistance in parasitic helminths; here, the application of proteomics is likely to be timely, given the increasing global threat of anthelmintic resistance to the food security sector [41]. Proteomic studies have begun to describe the action of antiparasitic drugs as well as to identify potential new drug targets.

**Figure 1:**
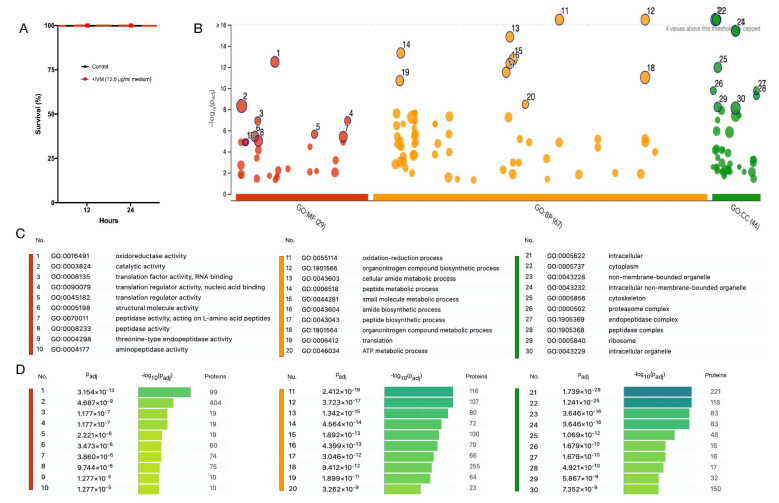
Graphical representation of obtained data. (**A**) Survival curve illustrates the percent of L3 larvae of *Anisakis simplex* s. s. which survived the ivermectin treatment. (**B**) Manhattan plot that illustrates the enrichment analysis results. The functional terms are grouped and color-coded by data sources, i.e., molecular function (MF) is **red**, biological processes (BP) is **orange**, and cellular components (CC) is **green**. (**C**) Ten top subcategories from each category are marked by a number and described. (**D**) The adjusted enrichment *p*-values in negative log_10_ scale with the number of proteins assigned to each subcategory. Detailed representation and annotation of all proteins submitted to the analysis can be found in Supplementary File 3.

In this study we identified 3433 proteins (File S1). After limiting the list to the proteins with at least two protein-unique peptides, we obtained 1247 proteins that were analyzed further (File S2). After IVM treatment at a concentration of 12.5 µg/mL of culture medium, all of the parasites were alive after 12 and 24 h of in vitro culture (Figure 1A).

### 3.1. Functional Enrichment Analysis

Based on the GO annotations, the proteins with at least two protein unique peptides (1247) were classified into three different categories: molecular function (MF, 29 different functions), biological processes (BP, 67 different processes), and cellular components (CC, 46 different components) (Figure 1B–D; File S3). The top 10 (a = 0.05) subcategories assigned for each of three main GO annotations are presented (Figure 1B–D). The functions assigned to the MF category were, with predominant catalytic activity groups, oxidoreductase activity (GO:0016491; 99 proteins), peptidase activity (GO:0070011, GO:0008233; 149 proteins), and aminopeptidase activity (GO: 0004177; 10 proteins) (Figure 1C,D). In the BP category, most of the proteins were involved in the oxidation‒reduction processes (GO:0055114; 116 proteins), as well as in the organonitrogen compound biosynthetic process (GO:1901566; 107 proteins) and organonitrogen compound metabolic process, but with a higher adjusted *p*-value (GO:1901564; 255 proteins) (Figure 1C,D). The distribution of the identified proteins according to their abundance in the cellular components was as follows: most of the proteins were associated with intracellular structures (GO:0005622; 221 proteins), the majority of them were predicted to be localized in the cytoplasm (GO:0005737; 118 proteins) and non-membrane-bound organelles (GO:0043228; 83 proteins) or cytoskeleton (GO:0005856; 48 proteins) (Figure 1C,D) (detailed description in File S3).

### 3.2. Differentially Regulated Proteins

**Figure 2:**
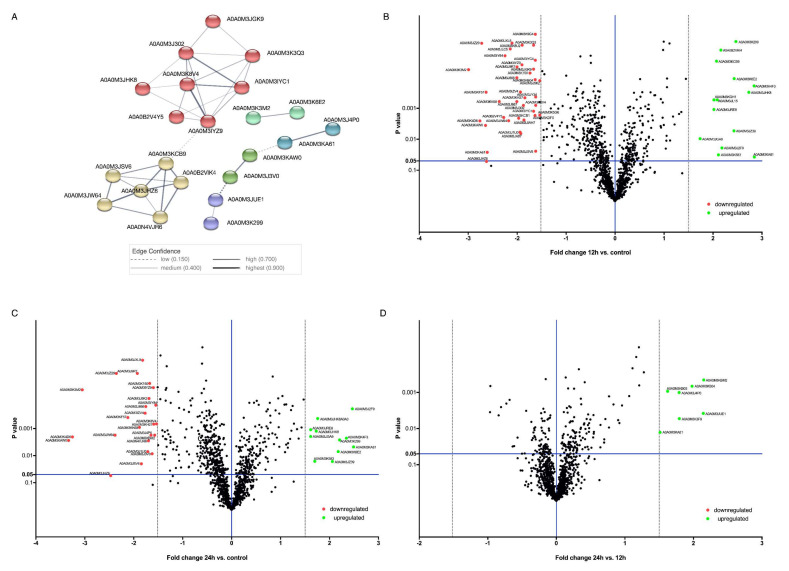
Graphical representation of obtained data. (**A**) Protein–protein interaction network analysis of differentially regulated proteins after ivermectin treatment of L3 larvae of *Anisakis simplex* s.s. Volcano-plot representations of the statistical analysis of ivermectin-treated L3 larvae of *Anisakis simplex* s.s.: (**B**) control vs. 12 h of treatment, (**C**) control vs. 24 h of treatment, and (**D**) 24 h of treatment vs. 12 h of treatment. To see the legend for protein IDs, see Supplementary File 2.

After relative quantification, the obtained results were further analyzed to obtain the final list of differentially regulated proteins (DRPs). Kruskal‒Wallis one-way analysis of variance was applied to detect significantly modulated proteins when comparing different abundance rations: control vs. 12 h, control vs. 24 h, and 24 h vs. 12 h (FC = 1.5; *p*-value ≤ 0.05) (File S2). Volcano plot representations of DRPs are shown in Figure 2B‒D. In all the presented volcano plots, the most upregulated proteins were towards the right, the most downregulated proteins were towards the left, and out of them the most statistically significant proteins were towards the top.

Presented volcano plots suggested more differences between the control and IVM treatment for 12 and 24 h (Figure 2B,C) and less evident differences between 24 and 12 h of IVM treatment (Figure 2D). After 12 and 24 h of IVM treatment of *A. simplex* s.s., we can assume that the response of the parasite to the drug was similar and the differences concerned only the number of proteins. Comparative proteomics analysis indicated that 59 proteins were differentially regulated (DRPs) in IVM-treated larvae, of which 14 were upregulated and 38 were downregulated after 12 h of in vitro culture, whereas 12 proteins were significantly upregulated and 22 were significantly downregulated after 24 h of IVM treatment (FC = 1.5; *p*-value ≤ 0.05) (Figure 2B–D; File S2). In this cohort, examples of the downregulated proteins, comparing 12 and 24 h of culture to the control, were aminopeptidase (A0A0M3J667), carboxypeptidase (A0A0M3K8V4), serine proteases (A0A0M3J5K2, A0A0M3IYC1, A0A0M3J9R7), metalloendopeptidase (A0A0M3KF57), cuticle collagen dpy-5 (A0A0M3KAW0), and cuticulin-1 (A0A0M3K3M2) (Figure 2B–D; File S2). At the same time, the upregulation of myosin tail 1 domain-containing protein (A0A0M3JHK8), endochitinase 1 (A0A0M3K6E2), lipase 3 domain-containing protein (A0A0M3K4F3), putative Kunitz-type protease inhibitor (A0A0M3KBM2), ShKT domain-containing protein (A0A0M3KA81), and TIL domain-containing protein (A0A0M3JGA9) was noted. Additionally, seven proteins were upregulated in the larvae cultured with IVM for 24 h in comparison with those cultured for 12 h (Figure 2D).

Summarizing, IVM reduced the presence of proteins responsible for the catabolism of amino acids, important during the process of parasite invasion and development, as well as molting, but increased the presence of proteins responsible for energy metabolism, motor proteins, and host immunomodulation.

### 3.3. Protein—Protein Interactions

Protein interactions network analysis was performed by submitting only DRPs (59 proteins) to the STRING (v. 10.5; ELIXIR, Hinxton, Cambridgeshire, UK). The analysis demonstrated a strong interaction network (Figure 2A). A total of 22 proteins constituted a very complex and strongly interactive network, which was clustered to a specified MCL inflation parameter (MCL = 3). Proteins not shown in Figure 2A were not connected with any other or not shown in the analysis results at all. This mainly reflects the fact that those interactions have been identified on the background of *C. elegans,* a free-living species, not a parasitic one like *A. simplex* s.s. These difficulties were described previously by Stryiński et al. [33].

According to MCL clustering, six nodes were obtained, each of them depicted as a group of different colored circles. Most of the analyzed proteins interacted with other proteins in complex protein‒protein interaction networks (Figure 2A). Among them, 37 interactions were shown in connection with co-expression, co-occurrence, and due to the appearance of any information on the interactions between those proteins in different databases (Figure 2A). The most complex nodes of those interaction networks were those related to energy metabolism, peptidase activity, oxidative metabolism, and cuticle creation, which in turn is closely connected with larval molting and parasite invasion. Analyzing only DRPs, the expected number of edges according to String was two, and the current set of proteins showed 37, which means that these proteins have more interactions among themselves than would be expected for a random set of proteins of similar size drawn from the genome. According to Stryiński et al. [33], this means that function-blocking or silencing of genes of these proteins (especially those with the highest number of interactions, e.g., carboxypeptidase, A0A0M3K8V4, or glutamate dehydrogenase, A0A0M3JHZ6) could be the basis for the disruption of metabolic processes important for the parasite, and such proteins could be good new targets for antiparasitic drugs.

### 3.4. The Transcription Levels of Selected DRPs

**Figure 3:**
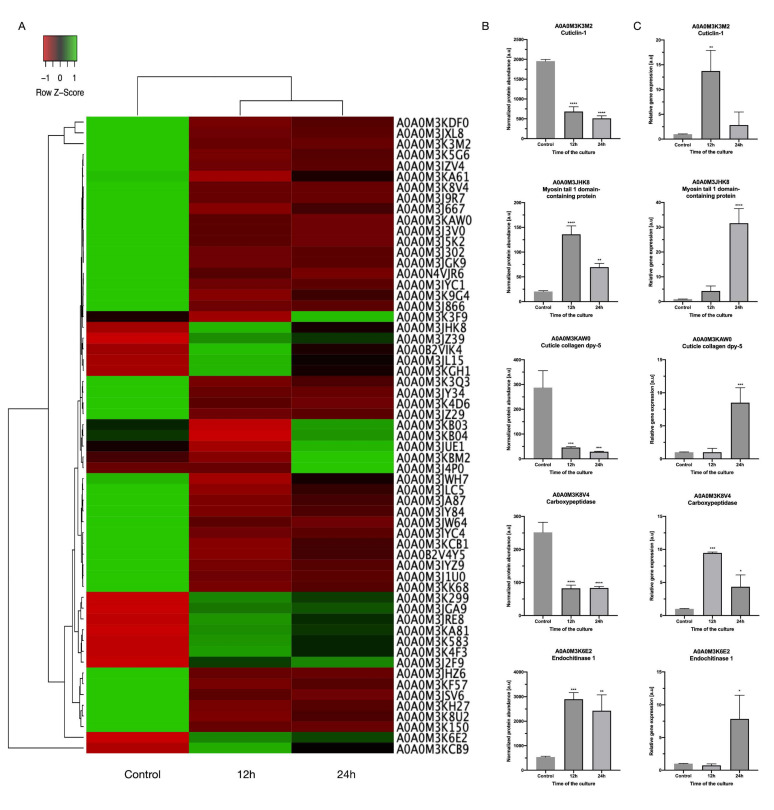
(**A**) Heat-map of all differentially regulated proteins (DRPs). (**B**) The protein abundance profiles of selected DRPs. Presented values were normalized between the samples and are originally from LC-MS/MS analysis. (**C**) The transcription levels of selected genes encoding chosen DRPs. The control is shown as normalized to a value 1, and the samples indicate the changes relative to the control. *p*-values below 0.1234 (non-significant) were considered statistically significant, where 0.0332 (*), 0.0021 (**), 0.0002 (***), and <0.0001 (****).

To validate and further assess the differential abundance of DRPs (Figure 3A,B), five of them were chosen, and the transcription levels of their genes were determined by real-time PCR (Figure 3C). Endochitinase 1 and myosin tail 1 domain-containing protein were selected as the upregulated proteins, whereas cuticle collagen dpy-5, carboxypeptidase, and cuticulin-1 were selected as the downregulated proteins in IVM-treated vs. untreated larvae.

The results of real-time PCR showed that the mRNA levels of two genes (endochitinase 1 and myosin tail 1 domain-containing protein) were approximately consistent with the protein abundance levels. However, the mRNA levels of carboxypeptidase, cuticle collagen dpy-5, and cuticulin-1 genes were inconsistent with the protein abundance levels. The contradiction between protein abundance and mRNA levels might be due to post-translational control, in which negative regulatory factors are possibly activated in the translation process, or the intrinsic mRNA stability is regulated by specific molecules such as microRNA [42]. However, the study’s defined significance levels were similar to those obtained for the five proteins in the shotgun proteomics analysis (Figure 3B,C). Significant differences were found, in that *p*-values ≤ 0.0001 (****) were observed for one gene, myosin tail 1 domain-containing protein. Less significant differences, *p*-value ≤ 0.0002 (***), were obtained in the case of two genes, cuticle collagen dpy-5 and carboxypeptidase, and differences where the *p*-value was ≤0.0332 (*) or ≤0.0021 (**) were observed for cuticlin-1 and endochitinase 1.

### 3.5. Detailed Description of Selected DRPs

Comparative proteomics analysis displayed that 59 proteins were differentially regulated in IVM-treated larvae. Some of them seem to be important in connection with efficient drug action or drug resistance.

Myosin is a motor protein that is essential for a variety of processes ranging from intracellular transport to muscle contraction [43]. Literature data and our results show that antiparasitic drugs, e.g., ivermectin or arctigenin [44], downregulate the protein level of myosin over time (viz., a significant reduction of protein level from 12 to 24 h of the culture), but compared to the control without IVM, the level of myosin was upregulated (Figure 3B). Additionally, *Fasciola hepatica*’s susceptibility to the anthelminthic drug triclabendazole, characterized by lethal activity, was indicated by the presence of actin, also one of the motor proteins. Moreover, the interaction of filamentous actin with myosin forms the basis of muscle contraction, which, when disrupted or taken over by antiparasitic drugs, might be the cause of parasite death [45,46]. According to the literature data mentioned previously, anthelmintics influence the motor proteins and thus reduce parasite motility, causing death; however, in our case, IVM did not affect the motility and survival of *A. simplex* s.s., even though the protein level of myosin was modulated. We believe that this may be due to the parasite’s defense mechanisms in the early hours of the antiparasitic treatment.

Furthermore, our results showed that some peptidases were downregulated in IVM-treated larvae, including metalloendopeptidase (A0A0M3KF57) and carboxypeptidase (A0A0M3IZV4). Moreover, in transcriptomic studies, up to 40% of transcripts identified in the pharynx of *A. simplex* s.s., compared to transcripts present in the pharynx of *Hysterothylacium aduncum*, a nonpathogenic nematode, were upregulated; most of them are from protein families linked to enzymatic activity of peptidases, like zinc metalloproteinase nas-13, and others such as lipases and serine/threonine‒protein kinases [47]. In addition, carboxypeptidase is necessary for new cuticle generation and body morphogenesis in free-living and parasitic nematodes; it is located at the hypodermis and interacts with other collagen-modifying enzymes [48]. We found that some collagen proteins (A0A0M3JZ29, A0A0M3K3M2, A0A0M3KAW0) were downregulated after 12 h of culture with IVM, and some cuticle collagen proteins like collagen cuticle N domain-containing protein (A0A0M3J3V0) were expressed only after 24 h of the IVM treatment. Our study showed that cuticlin abundance was reduced after 24 h of IVM treatment compared to 12 h. However, after 12 or 24 h of treatment, compared to the control, the protein abundance of cuticlin was decreased (Figure 3B). In addition, our study showed that collagen’s abundance was decreased compared to the control. This may be due to the parasite’s first line of defense, which is the cuticle. The drug diffusion is probably related to the morphological/functional properties of the parasite’s external surfaces. The direct effect of IVM on cuticle proteins was not described previously, but our results may be the first evidence of the effect of antiparasitic drugs on the parasite cuticle.

Additionally, endopeptidases, like the serine proteases detected in this study, play a significant role in the lifecycle of the parasite and in the pathogen‒host relationship that could be related to virulence, invasion of host tissues, and/or intracellular digestion (File S2) [49,50]. Moreover, the previously mentioned putative resistance of *Fasciola hepatica* to triclabendazole, characterized by sublethal activity, was indicated by the presence of cathepsin L, a peptidase [46].

The studies confirming this research show that downregulation or inhibition of putative endopeptidase bli-4 expression might be beneficial for parasite (*Trichinella spiralis*) survival and growth under albendazole stress, which might be helpful for a parasite to adjust to drug stress [51]. This protein has serine-type endopeptidase activity and is involved in several processes, including cellular response to salt, collagen and cuticulin-based cuticle development, and positive regulation of synaptic growth at the neuromuscular junction. In *A. simplex* s.s., downregulation of peptidase-type proteins during IVM treatment is probably an important strategy to survive antiparasitic treatment.

Furthermore, some of the DRPs obtained have been considered as potential vaccine candidates and drug targets for treating helminthiases, e.g., endochitinase 1, myosin, Kunitz inhibitor protease, or TIL domain-containing protein (File S2) [51,52,53,54]. Additionally, Kunitz inhibitor protease, trypsin inhibitor-like protein (TIL), and ShKT domain-containing protein could play important roles in the immunomodulation of host antiparasitic immunological responses. The Kunitz-type trypsin inhibitor (Ani s1) has an antigenic role among excretory‒secretory products [55,56]. The ShKT domain, which has also been called a nematode six-cysteine (NC6) domain [57] and ion channel regulator (ICR) [58] in venomous creatures, has likely been modified to give rise to potent ion channel blockers. The incorporation of this domain by roundworms into prolyl hydroxylases, astacin-like metalloproteases, and trypsin-like serine proteases could produce enzymes with potential channel-modulatory activity, which would be important in pathogenicity in the case of parasitic nematodes [59].

Intriguingly, in the present study we found that IVM did not significantly modulate xenobiotic-metabolizing proteins. Proteins such as glutathione S-transferases, xenobiotic reductases, and cytochromes P450 modulate the biological activity and behavior of many drugs, including anthelmintics. The effects of anthelmintics can often be abolished by these enzymes when the drugs are metabolized into an inefficient compound [60]. The dose of the drug is also important; knowing that different concentrations of IVM influence different types of receptors [14], the high concentration tested here (µmol) did not stimulate the expression of proteins responsible for the antioxidant response and, interestingly, did not cause higher mortality of larvae (Figure 1A).

Our results are inconclusive, testifying to the nonsusceptibility of *A. simplex* s.s. to IVM, as evidenced by the over-regulation or modulation of both proteins and transcripts, and larvae survival after IVM treatment. It appears that the role of IVM is limited, in this case, to being a modulator of the immune response and being involved in the cross-talk between parasite and host.

## 4. Conclusions

The most important findings, in our opinion, of this study are that *A. simplex* s.s. L3 larvae might be less sensitive to IVM and that this drug is not efficient at this larval stage and/or in this species. We think that the findings presented in our study could have important implications for using ivermectin against *A. simplex* s.s. and will contribute to a better understanding of the changes in parasite metabolism under the influence of this drug. To summarize, the present study shows the list of proteins of the L3 larval stage of *A. simplex* s.s. modulated by IVM. Interestingly, xenobiotic-metabolizing proteins were not identified as significantly modulated when comparing IVM-treated vs. control samples. Our results are the first to give extensive proteomic information on the response of *A. simplex* s.s. to IVM. A total of 59 differentially regulated *A. simplex* s.s. proteins were identified by LC-MS/MS analysis. Some of the proteins are primarily involved in cuticle synthesis, maintenance, remodeling, and degradation, as well as in hormonal regulation of molting and intracellular trafficking of molecular compounds and regulators. IVM does not have an impact on *A. simplex* s.s. motility and survival but, in our estimation, suppresses the parasite’s ability to evade the host’s natural immune defense mechanisms, which was confirmed by the list of differentially regulated proteins.

To gain more biological and clinical significance, our findings must be further validated in a functional model of Anisakis infection.

## Supplementary Materials

The following are available online at https://www.mdpi.com/2073-4425/11/6/710/s1. Figure S1: The visualization of SDS-polyacrylamide electrophoresis. Protein extracts were separated and evaluated on 12% (v/v) polyacrylamide gels (20:1) with a stacking gel of 4% polyacrylamide. Gel was silver stained. Lane 1—molecular weight marker; lanes 2–4—control samples; lanes 5–7—IVM-treated samples for 12 h; lanes 8–10—IVM-treated samples for 24 h; File S1: Data repository with all identified proteins for *Anisakis simplex* s.s. proteins cultured with and without ivermectin for 12 h and 24 h; File S2: Data repository with all differentially regulated proteins (DRPs) in *Anisakis simplex* s.s., cultured with and without ivermectin for 12 h and 24 h. Additionally, all proteins identified with at least two protein-unique peptides are presented; File S3: Data repository with detailed functional enrichment analysis., Table S1: The primers of ivermectin-induced DRPs used for real-time PCR assay for verification of mRNA levels in *Anisakis simplex* s.s. invasive larvae.
