# Supplementary material for: Comparative Proteomics Analysis of Anisakis simplex s.s.—Evaluation of the Response of Invasive Larvae to Ivermectin
Source: Genes (Basel). 2020 Jun 26;11(6):710. doi: 10.3390/genes11060710 (PMC7349835; doi:10.3390/genes11060710)
Supplement: Supplementary file 1 [file genes-11-00710-s001.zip › Polak et al. Table S1.docx]

Table 4. The primers of ivermectin-induced DRPs used for Real-time PCR assay for verification of mRNA levels in *A. simplex* s. s. invasive larvae.

| Gene name | Primer sequence (5’-3’) |  |  |
| --- | --- | --- | --- |
| Carboxypeptidase  Cuticle collagen dpy-5  Myosin domain-containing protein  Cuticlin-1  Endochitinase-1  Elongation factor 1-alpha | For. GATCCACCAGTGGGCTCAAA  For. TGCCGTTATAATTGCGTCGT  For. ATCAGGCACAAGTCGAGGAA  For. TGCATGTCTCCAATGGGCTT  For. CAACCACCAAAACCCGCAAT  For. TCCTCAAGCGTTGTTATCTGTT | | Rev. CCCTTTCGACGCTTCCAGAT  Rev. CGTGCGAGATCAGTTCAGAG  Rev. CCAGGTCTTGCATACGTTCG  Rev. CGCTGACGCATGAACGAAAT  Rev. CTTTGATGCGACGGCTTTCC  Rev. AGTTTTGCCACTAGCGGTTCC |
