# Supplementary figures and images for: Comparative Proteomics Analysis of Anisakis simplex s.s.—Evaluation of the Response of Invasive Larvae to Ivermectin
Source: Genes (Basel). 2020 Jun 26;11(6):710. doi: 10.3390/genes11060710 (PMC7349835; doi:10.3390/genes11060710)

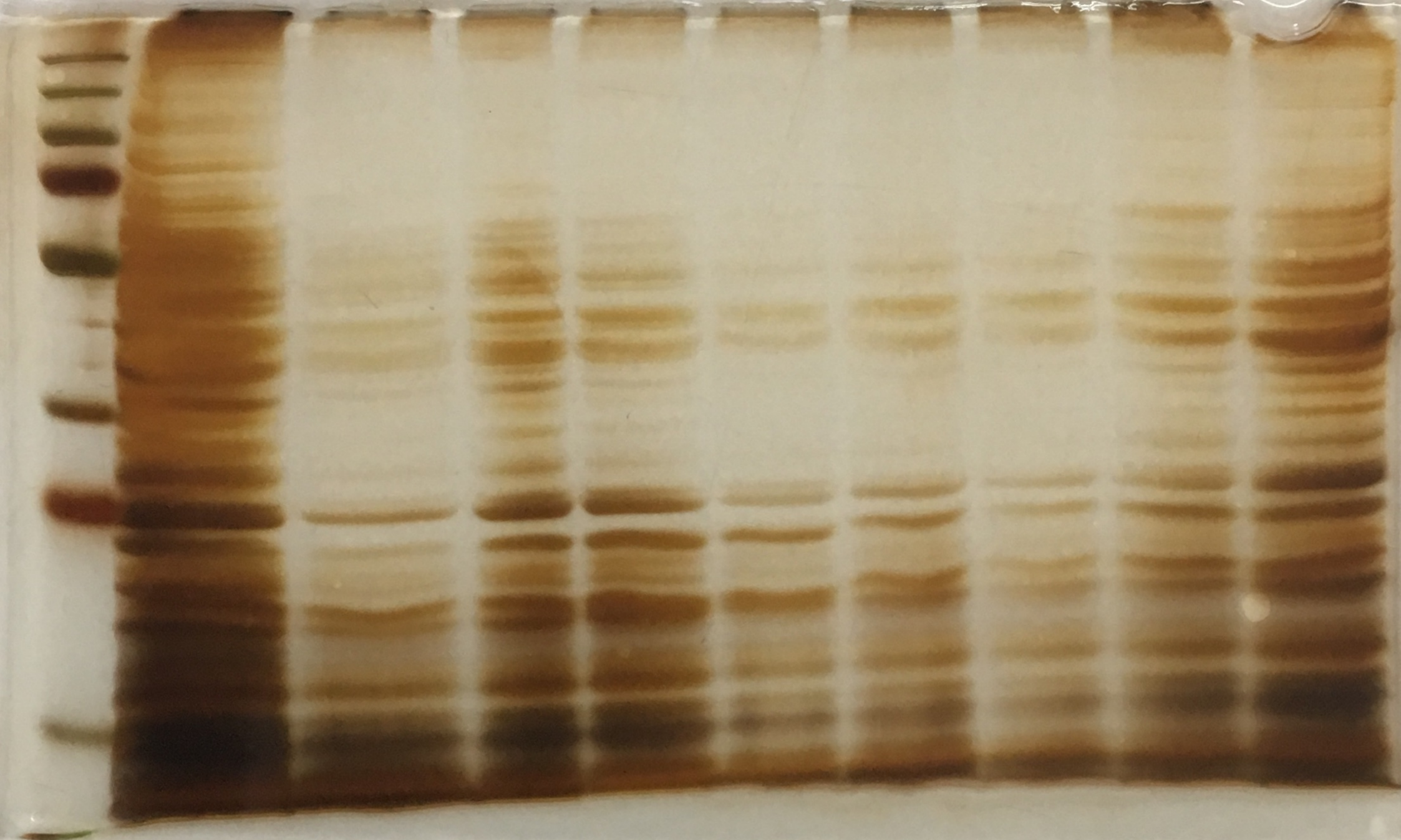

Supplement: Supplementary file 1 [file genes-11-00710-s001.zip › Polak et al. Figure S1.pdf]
